# Platelet Mitochondrial Function, Physical Performance, and Body Composition in Older People Living with HIV: A Preliminary Study

**DOI:** 10.3390/ijms27093972

**Published:** 2026-04-29

**Authors:** Rosemary A. Schuh, Sausan M. Jaber, Krisann K. Oursler, Alice S. Ryan

**Affiliations:** 1 VA Research Service, Baltimore VA Medical Center, Baltimore, MD 21201, USA; 2 Baltimore VA Medical Center Geriatric Research, Education and Clinical Center (GRECC), Baltimore, MD 21201, USA; sausan.jabertaha@va.gov; 3 Division of Infectious Disease, Department of Medicine, Virginia Tech Carilion School of Medicine, Roanoke, VA 24016, USA; oursler@vt.edu; 4 Salem Veterans Affairs Health Care System, Salem, VA 24153, USA; 5 Division of Geriatric and Palliative Medicine, Department of Medicine, School of Medicine, University of Maryland, Baltimore, MD 21201, USA

**Keywords:** mitochondrial bioenergetics, platelet mitochondrial function, aging in HIV, physical performance, body composition, SIRT3, cardiorespiratory fitness, exercise

## Abstract

Mitochondrial dysfunction is a hallmark of aging and age-related physical decline in people living with HIV (PLWH) who experience accelerated aging. This pilot study investigated the relationships between platelet mitochondrial function, physical performance, and body composition in older, sedentary PLWH compared with older, sedentary HIV-negative controls. Platelets have the potential to act as minimally invasive and easily accessible biomarkers for systemic mitochondrial bioenergetics and may serve as a practical biomarker in aging-related research. We analyzed correlations between mitochondrial parameters, protein levels, and measures of physical performance and body composition in a cohort of predominantly African American men (*n* = 7 PLWH, *n* = 7 controls). Body composition was assessed using dual-energy X-ray absorptiometry (DXA), and exercise capacity through VO_2_ peak and strength tests. Platelet mitochondrial bioenergetic parameters were measured by oxygen consumption rates (OCR) and extracellular acidification rates (ECAR). Key mitochondrial proteins SIRT3, COXII, DRP1, and OPA1 were evaluated by Western blotting. The PLWH and HIV-negative control groups were similar in age and cardiorespiratory fitness. In PLWH, basal OCR and ATP-linked respiration showed strong positive correlations with VO_2_ peak (r = 0.874, *p* < 0.05 and r = 0.862, *p* < 0.05, respectively) and negative correlations with BMI (r = −0.856, *p* < 0.05 and r = −0.849, *p* < 0.05, respectively). SIRT3 emerged as a potential key player, demonstrating strong positive correlations with basal OCR (r = 0.804, *p* < 0.05), ATP-linked respiration (r = 0.787, *p* < 0.05), and VO_2_ peak (r = 0.970, *p* < 0.001), and negative correlations with BMI (r = −0.830, *p* < 0.05) and fat mass (r = −0.827, *p* < 0.05) in PLWH. Analyses focused on within-group associations in PLWH because bioenergetic measures were obtained using different Seahorse platforms in PLWH and controls, precluding valid direct quantitative comparisons between groups. Our findings provide evidence for significant associations between platelet mitochondrial bioenergetics, specific mitochondrial proteins (particularly SIRT3), and key physical attributes in older, sedentary PLWH. These preliminary findings suggest that platelets may serve as minimally invasive biomarkers of systemic mitochondrial health, contribute to our understanding of mitochondrial function in HIV-associated accelerated aging, and inform future interventions to enhance mitochondrial function and improve health outcomes in this vulnerable population. However, results should be interpreted cautiously given the small sample size and exploratory design and should be considered hypothesis-generating rather than definitive. Larger, demographically more diverse studies that include HIV-negative controls are needed to validate these associations and determine their clinical relevance.

## 1. Introduction

Aging is accompanied by physiological changes that increase susceptibility to cardiovascular disease, metabolic disorders, and physical decline [1,2,3,4]. In people living with HIV (PLWH), these age-related risks are compounded by chronic immune activation, persistent inflammation, and the long-term effects of antiretroviral therapy [3,5,6,7]. Despite effective ART and viral suppression, PLWH exhibit a higher burden of comorbidities such as cardiovascular disease, diabetes, and osteoporosis at earlier ages compared to HIV-negative individuals [8,9,10]. This phenomenon, often described as “accelerated aging,” may make PLWH an informative model for studying aging processes [11,12,13].

HIV infection induces chronic inflammation and immune activation, which contribute to the development of aging-related comorbidities and mirror many features of biological aging, such as cellular senescence, mitochondrial dysfunction, and dysregulation of metabolic processes [14,15,16,17]. ART, while effective in controlling viral replication, has been linked to mitochondrial toxicity, contributing to cellular dysfunction and systemic metabolic disturbances that may further accelerate aging processes [17,18,19]. Therefore, PLWH are particularly vulnerable to mitochondrial dysfunction, making them an important population for studying mitochondrial health and bioenergetics.

A promising and non-invasive way to study mitochondrial health in aging PLWH is through platelets. Platelets, usually recognized for their role in hemostasis, are increasingly understood to be key players in inflammation, immune responses, and cardiovascular pathology, particularly as they age [20]. Platelets continuously circulate systemically and are exposed to signals of systemic inflammation, metabolic stress, and cardiovascular risks. This exposure suggests that their mitochondrial phenotype may reflect broader bioenergetic alterations caused by aging. Aging platelets exhibit impaired bioenergetic function and increased oxidative stress, which can contribute to a pro-thrombotic and pro-inflammatory state, thus accelerating cardiovascular disease progression [21,22]. Studying mitochondrial dysfunction in platelets offers a minimally invasive method to assess systemic bioenergetic health, as mitochondrial changes in platelets have been proposed as a link between inflammation, oxidative stress, and cardiovascular risk, especially in older adults [23,24,25,26].

Mitochondrial sirtuins (SIRT3-5) are critical mediators of metabolic plasticity, with SIRT3 being the primary nicotinamide adenine dinucleotide (NAD+)-dependent deacetylase localized to the mitochondrial matrix [27]. SIRT3 activity is reduced by lower NAD+ levels, leading to the acetylation of its target proteins, which regulates key processes in energy metabolism, including tricarboxylic acid cycle (TCA) enzymes, electron transport chain (ETC) complexes, reactive oxygen species (ROS) degradation, and mitochondrial dynamics such as biogenesis, fission, and fusion [27,28]. In healthy populations, SIRT3 supports mitochondrial substrate metabolism, protection against oxidative stress, cell survival, and longevity [29]. Evidence suggests that SIRT3 expression declines with age and contributes to frailty and metabolic dysfunction. Investigating platelet SIRT3 in older PLWH may reveal a potential target linking mitochondrial health to physical performance [30].

Mitochondrial morphology is maintained through a balance between fragmentation (fission) and the formation of elongated structures (fusion), regulated by proteins like dynamin-related protein 1 (DRP1) and optic atrophy protein 1 (OPA1). Abnormalities in these processes, particularly in PLWH, are associated with aging-related outcomes, including loss of muscle and frailty [31,32,33].

In PLWH, chronic immune activation and inflammation from HIV infection can further disrupt mitochondrial dynamics, impairing processes like fission, fusion, and mitophagy, which are essential for maintaining mitochondrial function and preventing cellular damage [17,34]. Mitochondrial proteins such as SIRT3, COXII, DRP1, and OPA1 are vital for regulating energy production, oxidative stress, and cellular homeostasis [29,35]. These disruptions are exacerbated in older PLWH, contributing to frailty, reduced physical function, and increased comorbidities [17,36]. Mitochondrial dysfunction is central to cellular metabolism and ATP production, and its decline is linked to decreased physical performance, sarcopenia, and frailty in aging populations [37,38,39,40]. The expression of sirtuins, including SIRT3, declines with age, which may further impair oxidative stress regulation and energy metabolism [35,41]. Likewise, mitochondrial fusion and fission proteins such as DRP1 and OPA1 are critical for mitochondrial integrity, and their dysfunction is associated with aging and related diseases [23,32,42,43,44]. In PLWH, the chronic inflammatory state may further accelerate these mitochondrial impairments, exacerbating aging-related mitochondrial dysfunction [5,8,45,46,47].

Physical function and exercise capacity are strongly associated with mitochondrial health in older adults [40]. Reduced mitochondrial respiratory capacity has been correlated with decreased muscle strength, impaired exercise tolerance, and increased frailty in aging populations [40,48,49]. Given the central role of mitochondria in energy metabolism, exploring mitochondrial function in platelets may provide insights into systemic bioenergetic health, particularly in older PLWH, who experience both HIV-related mitochondrial dysfunction and age-associated declines in mitochondrial efficiency [4,11,13,47,50].

Mitochondrial activity has been measured in different tissues and cell types utilizing various methodologies. Prior studies, including our own, have assessed mitochondrial health in skeletal muscle (citrate synthase, mitochondrial mass) or ETC components (mitochondrial activity) [51,52]. Less invasive protocols have been established utilizing components of blood that can be readily acquired. These minimally invasive procedures have led to in-depth studies investigating mitochondrial function in chronic diseases as well as traumatic injury including spinal cord injury [51,53,54]. Platelets have few mitochondria per cell, but established protocols allow large numbers of platelets to be isolated, providing samples with relatively high mitochondrial content for respirometry analyses [53,55].

This pilot study investigates the mitochondrial bioenergetic profile of platelets in older, sedentary PLWH and examines correlations between mitochondrial protein expression, bioenergetic function, and physical performance. We also enrolled an age-, sex-, and lifestyle-matched (sedentary) HIV-negative control group primarily to provide a descriptive benchmark for physical performance and body composition; however, due to methodological constraints (different Seahorse platforms), quantitative comparisons of platelet bioenergetics between PLWH and controls were not performed. While previous studies have primarily focused on group-level comparisons, the present study emphasizes within-cohort associations between platelet mitochondrial function and functional outcomes, underscoring the potential utility of platelets as integrative biomarkers of systemic bioenergetic health. By analyzing key mitochondrial markers such as SIRT3, COXII, OPA1, and DRP1, the study seeks to elucidate the relationship between platelet mitochondrial health and overall physical function in this aging population. We hypothesize that platelet mitochondrial dysfunction may reflect broader systemic bioenergetic impairments that contribute to reduced physical performance and metabolic abnormalities in aging PLWH. Findings from this pilot study may guide follow-up research on specific ETC components that play a critical role in energy utilization in aging PLWH.

## 2. Results

Cohort demographics and characteristics are presented in Table 1. Sixty-four percent of the participants were over 60 years old, and approximately 86% were overweight or obese (BMI ≥ 25.0 kg/m^2^).

**Figure 1:**
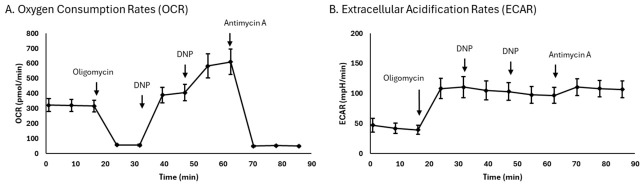
Representative OCR (**A**) and ECAR (**B**) by platelets isolated from blood. Oligomycin (2.5 μM), DNP (15 μM), and antimycin A (10 μM) were added when indicated. Data are presented in absolute rates as mean ± standard deviation (*n* = 7). Abbreviations: DNP, 2,4, dinitrophenol; ECAR, extracellular acidification rate; Basal ECAR, basal measurement prior to oligomycin addition; Max ECAR, maximal ECAR measurement prior to DNP addition; GR, glycolytic reserve, difference between basal and maximal ECAR; OCR, oxygen consumption rates.

Figure 1 graphically represents mean oxygen consumption rates (OCR, Figure 1A) and mean extracellular acidification rates (ECAR, Figure 1B) of the study cohort.

Platelet bioenergetic measurements, mean mitochondrial OCR, ECAR, and protein levels (SIRT3, COXII, DRP1, OPA1) are presented in Table 2. However, because bioenergetic measurements were obtained using different Seahorse platforms, these values should be interpreted cautiously and considered descriptive rather than definitive quantitative comparisons; they may provide a qualitative reference for future investigations.

Our correlation analysis revealed significant relationships between mitochondrial bioenergetics, mitochondrial proteins, and physical performance measures in older, sedentary PLWH. Basal OCR and ATP-linked respiration showed strong positive correlations with VO_2_ peak. Specifically, basal OCR correlated with VO_2_ peak (r = 0.874, *p* < 0.05), while ATP-linked respiration correlated with VO_2_ peak (r = 0.862, *p* < 0.05). These results suggest that higher mitochondrial respiratory capacity is associated with better cardiorespiratory fitness in older PLWH. Interestingly, basal OCR, maximal OCR, and ATP-linked respiration showed negative correlations with BMI (r = −0.856, *p* < 0.05; r = −0.809, *p* ≤ 0.05; and r = −0.849, *p* < 0.05, respectively), indicating that individuals with higher basal mitochondrial respiration tend to have lower BMI.

**Figure 2:**
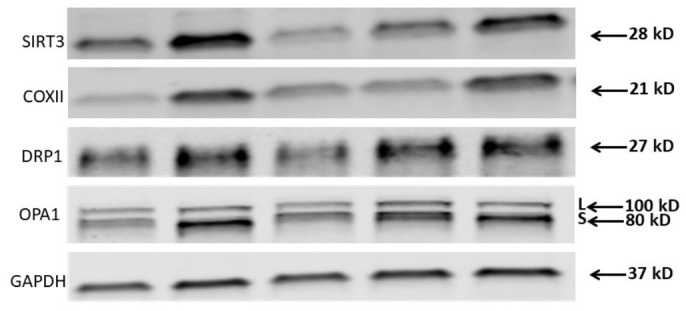
Representative Western blot images of COXII, SIRT3, DRP1 and OPA1 in platelets isolated from HIV-infected individuals. The OPA1 oligomeric complex comprises the long (L) fusogenic form and the short (S) form [56] (*n* = 5). GAPDH was utilized as a loading control. Abbreviations: COXII, cytochrome c oxidase; SIRT3, sirtuin 3; OPA1, optic atrophy protein 1 (fusion protein); DRP1, dynamin-related protein 1 (fission protein); GAPDH, glyceraldehyde 3-phosphate dehydrogenase.

**Figure 3:**
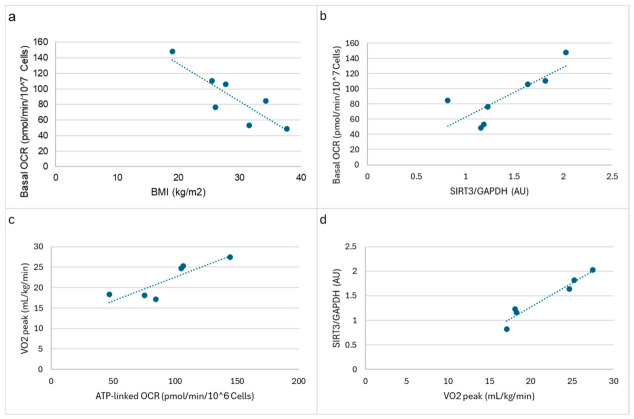
Representative associations between platelet mitochondrial function, SIRT3 expression, body composition, and aerobic capacity in older PLWH are shown in the following: (**a**) Relationship between body mass index (BMI) and platelet basal oxygen consumption rate (OCR); (**b**) Association between SIRT3 protein expression (normalized to GAPDH) and basal OCR; (**c**) Association between ATP-linked OCR and peak aerobic capacity (VO_2_ peak); and (**d**) Relationship between VO_2_ peak and SIRT3 expression.

Representative immunoblot images of SIRT3, COXII, DRP1, OPA1 and GAPDH are presented in Figure 2. Among the mitochondrial proteins examined within this limited PLWH cohort, SIRT3 emerged as a potentially important player, showing strong correlations with both bioenergetic parameters and physical performance measures. BMI correlated with platelet basal OCR (r = −0.856, *p* < 0.05, Figure 3a). SIRT3 correlated positively with basal OCR (r = 0.804, *p* < 0.05, Figure 3b) and ATP-linked respiration (r = 0.787, *p* < 0.05). ATP-linked OCR was associated with VO_2_ peak (r = 0.862, *p* < 0.05, Figure 3c). VO_2_ peak showed a robust positive correlation with SIRT3 (r = 0.970, *p* < 0.001, Figure 3d). Furthermore, SIRT3 correlated negatively with BMI (r = −0.785, *p* < 0.05) and fat mass (r = −0.827, *p* < 0.05). These findings demonstrate SIRT3’s potential role in regulating mitochondrial function and its association with favorable metabolic outcomes in this population. Other mitochondrial proteins (COXII, DRP1, and OPA1) showed varying degrees of correlation with bioenergetic parameters and physical attributes, though these relationships were generally weaker and often not statistically significant.

## 3. Discussion

People living with HIV (PLWH) have relatively normal life expectancy due to ART yet are at increased risk of early onset aging-related sequelae, including diabetes, cardiovascular disease, cancers, and neurocognitive decline [11,57]. Mitochondrial dysfunction leading to oxidative stress and cellular senescence is a feature associated with many of these same aging-related diseases [38,58,59]. Recent reviews have highlighted the challenges of translating basic scientific discoveries into clinically actionable tools for HIV care and aging-related complications [60]. Understanding mitochondrial dysfunction in PLWH is particularly important given its potential contribution to clinically significant outcomes such as sarcopenia, frailty, and cardiovascular disease in aging HIV populations. This pilot study contributes to the growing body of evidence suggesting that mitochondrial bioenergetics in PLWH may be compromised, particularly in terms of a greater reliance on glycolytic pathways for energy production, potentially as a compensatory mechanism in response to mitochondrial inefficiency [61].

HIV negative controls provide comparative benchmarks for physical function and body composition measures. While bioenergetic and protein data from this control group may not be directly compared, the inclusion of these participants offers an additional perspective on the functional status of older adults living with HIV. The strong positive correlations between basal oxygen consumption rate (OCR), ATP-linked respiration, and maximal oxygen consumption (VO_2_ peak) suggest that higher mitochondrial respiration is associated with better cardiorespiratory fitness in older PLWH. This aligns with previous research demonstrating the importance of mitochondrial function in maintaining aerobic capacity and overall physical performance.

Interestingly, we observed negative correlations between mitochondrial respiratory parameters and body mass index (BMI). This inverse relationship suggests that individuals with lower BMI tend to have higher basal, maximal, and ATP-linked respiration.

Among the mitochondrial proteins examined, SIRT3 emerged as a potentially important factor, showing strong associations with both bioenergetic parameters and physical performance measures. SIRT3’s positive correlation with VO_2_ peak and negative correlations with BMI and fat mass highlight its potential role in regulating mitochondrial function and influencing metabolic outcomes in older PLWH. These findings are consistent with previous research demonstrating SIRT3’s importance in maintaining mitochondrial health and metabolic homeostasis.

Mitochondrial targets of SIRT3 deacetylation include the antioxidant superoxide dismutase-2 and OPA1 [27,44]. Inhibition of SIRT3 results in hyperacetylation of these target proteins giving rise to oxidative stress and loss of mitochondrial fusion decreasing mitochondrial respiratory efficiency and facilitating apoptosis [29,43]. The present study in clinically stable, sedentary older PLWH suggests that mitochondrial function may be compromised in this cohort. These results align with growing evidence that mitochondrial dysfunction plays a key role in the aging process, especially in PLWH, who experience accelerated aging due to chronic immune activation and the long-term effects of antiretroviral therapy.

Several studies have examined mitochondrial functional endpoints utilizing peripheral blood mononuclear cells (PBMCs) [40,54,62]. In a small prospective study, OCR deficits in PBMCs isolated from PLWH were significantly ameliorated following an aerobic exercise regime [62]. A separate study in individuals with a sedentary lifestyle determined that PBMCs had improved bioenergetics and fatty acid oxidation following a low-intensity exercise regime [40]. Willig et. al., describe altered bioenergetic profiles in PBMCs isolated from women living with HIV that had high adiposity levels [54]. In one study in platelets, which focused on platelet-derived mitochondrial DNA (mDNA) content and examined mitochondrial OCR/ECAR determined that a small subset of PLWH had decreased platelet mDNA compared to age-matched controls and suggested this may result in reduced energy supply [16]. Thus, there remains a gap in the literature relating bioenergetics to functional status.

Our earlier study in skeletal muscle isolated from older PLWH demonstrated reduced mitochondrial oxidative enzyme activity, reduced ATP content, increased oxidative stress and 33% lower VO_2_ peak compared to age-matched controls that may contribute to reduced aerobic capacity [52].

Our findings add to the growing body of literature on platelet bioenergetics as a model for studying mitochondrial dysfunction in aging and chronic diseases. Given that PLWH experience accelerated aging, they may serve as a valuable model for understanding the complex interplay between mitochondrial dysfunction, metabolic alterations, and physical function in older adults.

Although our study cohort is limited in sample size and generalizability, the measurement of bioenergetics combined with mitochondrial proteins, aerobic capacity, body composition, and several measures of physical function in this population are additional dimensions that strengthen the novelty of this small study. An additional limitation of this study is the demographic composition of the cohort, which consisted exclusively of older males, predominantly African American. As a result, the findings may not be generalizable to women or younger individuals. One notable limitation of this study is its cross-sectional design, which makes it difficult to establish causal relationships between mitochondrial bioenergetics, physical function, and body composition. Additionally, the use of different bioenergetic platforms (XFe24 for PLWH and XFe96 for controls) and the lack of mitochondrial protein data in the control group limited our ability to perform direct statistical comparisons of mitochondrial function between groups. Future studies with larger sample sizes with similar mitochondrial measures are needed to further elucidate the relationships between mitochondrial function, SIRT3 expression, and physical performance in PLWH.

The implications of this pilot study suggest that targeting mitochondrial function, particularly through SIRT3 activation, may be a promising approach to mitigate age-related decline in PLWH. Further studies measuring SIRT3 and its target proteins could assess the utility of SIRT3 as a measure of mitochondrial health in blood samples from PLWH and potentially inform the development of interventions to enhance mitochondrial function and improve overall health outcomes in this vulnerable population [58].

## 4. Materials and Methods

### 4.1. Participants

Demographic and clinical characteristics were obtained at the baseline study visit through interviews and a review of medical records. Demographic variables included age, sex, race, and smoking status. Our patient demographics for this particular population are 99% male in our Veteran hospital. HIV disease status was characterized using CD4+ T-cell counts, a marker of immune function, and plasma HIV-1 RNA concentrations, which reflect active viral replication and viral control with antiretroviral therapy.

This clinical study included seven sedentary control and seven sedentary PLWH men ≥50 years of age. PLWH were recruited from the Baltimore VA Medical Center (VAMC) Infectious Disease Clinic, who were on a consistent ART regimen for at least 6 months. Exclusion criteria included myocardial infarction (within the previous 3 months), severe congestive heart failure (EF < 20% in previous year or NYHA Classification III or IV), uncontrolled hypertension (SBP > 180 and/or DBP > 110 mm Hg), peripheral vascular disease with claudication and use of medication affecting heart rate (e.g., β-blockers). Severe arthritis or neurologic disease limiting ambulation, participation in exercise (structured resistance or aerobic exercise > 1 time per week), and signs/symptoms of comorbidity that precluded exercise testing were also exclusionary. Detailed study eligibility criteria are provided in clinicaltrials.gov (NCT02101060).

### 4.2. Body Composition and Exercise VO_2_ Capacity

Body weight and height were determined to calculate body mass index (BMI). The study participants’ lean tissue and fat mass were determined with a total body scan by dual-energy x-ray absorptiometry (DXA) (Model GE iDXA). Peak oxygen consumption (VO_2_ peak), strength (1-repetition maximum (1-RM) for the leg press, leg curl, and seated row), and physical function (grip strength, timed repeated chair stands) were performed as previously described [63].

### 4.3. Whole Blood Draw and Platelet Isolation

Mitochondrial bioenergetic measurements were assessed in platelets isolated from fasted participants’ whole blood as described with modifications. Specifically, whole blood was centrifuged at 500× *g* for 10 min at 25 °C with acceleration (5) and deceleration (0) using a Beckman Coulter Allegra X-30R centrifuge (Brea, CA, USA) to obtain the platelet rich plasma (PRP) [64]. Aliquots of PRP were separated for platelet sedimentation to be utilized for Western blotting. These aliquots had the PPP removed, and the platelet pellet was resuspended in lysis buffer containing RIPA (5 mM Tris-HCl, pH 8.0, 15 mM NaCl, 0.05% deoxycholate, 0.1% NP-40, 0.1% sodium dodecycl sulfate) and protease inhibitor cocktail (Thermo Scientific, Waltham, MA, USA) and then immediately stored at −80 °C.

PRP was then centrifuged at 1600× *g* for 10 min at 25 °C to collect the platelet pellet. The pellet was resuspended in pre-warmed assay buffer containing Dulbecco’s modified EagleE medium (DMEM) supplemented with 1 mM pyruvate, 6.2 mM EDTA, 5.5 mM D-glucose and 4 mM L-glutamine (Thermo Scientific) at pH 7.4.

Platelets were counted on a Nexcelom Cellometer Vision Cell Counter (Nexcelom Bioscience, Lawrence, MA, USA) according to the manufacturer’s protocol. Briefly, cells were incubated with calcein-AM (Nexcelom Bioscience) at 37 °C in the dark for 20 min prior to loading on SD100 slides.

### 4.4. Platelet Bioenergetic Measurements

Platelets were attached to either V7 (XFe24) or V3 (XFe96) polystyrene plates (Agilent Technologies, Santa Clara, CA, USA), pre-coated with 3.5 ug/cm^2^ Corning^®^ Cell-Tak™ (Bedford, MA, USA) at a concentration of 3 × 10^7^ or 1 × 10^7^ per well, respectively. Platelet respirometry measurements were conducted utilizing the Seahorse Extracellular Flux Analyzer XFe24 (PLWH) or XFe96 (controls) (Agilent Technologies) as previously described [64]. For each participant, platelet bioenergetic measurements were performed in technical replicates of 8–16 wells per subject, depending on the machine used and the quantity of platelets isolated.

Oxygen consumption rates (OCR) were measured to determine basal, proton leak, ATP-linked, maximal, and non-mitochondrial respiration. Basal OCR is the rate necessary to sustain normal cellular energy demand. Proton leak is approximated by the oligomycin-insensitive OCR and represents wasted energy. ATP-linked respiration is the difference between basal respiration and respiration following the injection of the ATP synthase inhibitor oligomycin; it indicates OCR due to ATP synthesis via oxidative phosphorylation. Maximal respiratory capacity is measured following the injection of the uncoupler dinitrophenol; it is the theoretical maximum oxygen consumption [65]. Non-mitochondrial respiration is indicated by the antimycin A-insensitive OCR. Subtraction of the maximal from basal OCR is referred to as the reserve capacity, representing the maximal potential respiratory capacity the cell can utilize during increased energy demand. ECAR measurements included basal glycolysis (rate prior to oligomycin injection), maximal glycolysis (oligomycin-induced ECAR), and glycolytic reserve (difference between basal and maximal glycolysis), representing the capacity of the cell to upregulate glycolysis in response to increased energetic demand.

Platelet bioenergetic measurements were performed using Seahorse XFe24e analyzers (Agilent Technologies, Santa Clara, CA, USA) for PLWH samples and XFe96 analyzers (Agilent Technologies) for control samples due to instrument availability at the time of testing. Although both platforms utilize the same assay chemistry and analytical principles, absolute OCR and ECAR values differ between platforms. Therefore, bioenergetic data from PLWH and control participants were not intended for direct quantitative comparison. All primary analyses are restricted to within-group associations in PLWH.

### 4.5. Western Blotting

Protein levels for SIRT3, 1:1000 (Cell Signaling Technology, Danvers, MA, USA); cytochrome c oxidase subunit II (COXII, 1:4000, Abcam, Cambridge, MA, USA); dynamin-related protein1 (DRP1, 1:1000, fission protein, BD Biosciences, San Jose, CA, USA), optic atrophy protein (OPA1, 1:1000, fusion protein, BD Biosciences) and glyceraldehyde 3-phosphate dehydrogenase (GAPDH, 1:14,000, Cell Signaling Technology) in platelets isolated from the same blood draw as for the bioenergetics were resolved using precast Mini-Protean TGX any KD gels (Bio-Rad, Hercules, CA, USA) and transferred to a polyvinylidene difluoride membrane using a Trans-Blot Turbo transfer system (Bio-Rad). Blot images were collected on a Li-Cor Odyssey (Li-Cor Biosciences, Lincoln, NE, USA). All blots were probed simultaneously for the protein of interest, with GAPDH as the loading control. Densitometry analysis was performed using the Li-COR Image Studio Software (version 6.1). All target proteins were normalized to GAPDH. Due to limited platelet material from control participants, mitochondrial protein expression analyses were performed only in PLWH samples.

### 4.6. Statistics

Comparisons between groups were tested using Student’s *t*-tests. The Shapiro––Wilk test for normality was used to test data distribution. Pearson correlation (Spearman correlation if data distribution was not normal) and linear regressions were utilized to test associations between protein levels (SIRT3, COXII, DRP1, OPA1), bioenergetic rates, body composition, fitness and functional performance. All analyses were two-tailed and performed using SPSS (v. 30; Armonk, NY, USA), and significance was set at *p* < 0.05. Data are presented as means ± standard deviation.

## 5. Conclusions

In conclusion, this pilot study provides valuable insights into the relationships between platelet mitochondrial function, physical performance, and body composition in older, sedentary people living with HIV (PLWH). Our findings highlight the potential of platelet bioenergetics as a minimally invasive biomarker for assessing systemic mitochondrial health in this population. Key conclusions from our study include the strong correlation between mitochondrial respiratory capacity, as measured by basal oxygen consumption rate (OCR) and ATP-linked respiration, and cardiorespiratory fitness (VO_2_ peak) in older PLWH, suggesting that better mitochondrial function is associated with improved aerobic capacity. SIRT3 has been implicated in mitochondrial health, showing strong positive correlations with mitochondrial respiratory parameters and negative correlations with BMI and fat mass, underscoring its potential role in regulating mitochondrial function and influencing metabolic outcomes in PLWH. Given the limited sample size, this study is designed to generate hypotheses regarding potential relationships rather than to establish definitive conclusions. While SIRT3 demonstrated strong associations with mitochondrial and functional measures, these findings should be interpreted cautiously and require validation in larger cohorts. The observed associations between mitochondrial function, body composition, and physical performance support the hypothesis that mitochondrial dysfunction may contribute to accelerated aging in PLWH. The inclusion of an HIV-negative control group, although limited by methodological constraints, offers useful context to interpret the functional impairments observed in the PLWH cohort. However, the inability to directly compare bioenergetic or protein expression data between groups underscores the need for future studies. These preliminary findings contribute to our understanding of the role of mitochondrial function in HIV-associated accelerated aging and may inform future research on targeted interventions to enhance mitochondrial health in PLWH.

## Figures and Tables

**Table 1:** Study cohort demographics, characteristics, mitochondrial OCR/ECAR, and protein levels.

	Mean ± SD	Range	Mean ± SD	Range
Demographics	PLWH *n* = 7		Control *n* = 7	
Age (years)	63.1 (6.4)	55–73	61.4 (6.4)	52–71
Gender (Male: Female)	7:0		7:0	
Race (African American: Caucasian)	6:1		7:0	
Smoking (ever), *n* (%)	4 (57%)		---	
CD4, cell/mL	610.6 (245.9)	202–850	N/A	
HIV-1 RNA < 20 copies/mL, *n* (%)	7 (100%)		N/A	
Duration of HIV infection (years)	21.7 (8.4)		N/A	
Hypertension, *n* (%)	6 (86%)		6 (86%)	
Type 2 Diabetes, *n* (%)	5 (71%)		0 (0%)	
**Obesity**				
(BMI > 30 kg/m^2^), *n* (%)	4 (57%)		6 (86%)	
**Overweight**				
(BMI > 25 kg/m^2^), *n* (%)	5 (71%)		7 (100%)	
Hepatitis C antibody, *n* (%)	4 (57%)		0 (0%)	
**Characteristics**				
Weight (kg) **	87.3 (12.6)	73.9–105.9	105 (9.6)	87.5–115
BMI (kg/m^2^)	28.8 (6.2)	19–37.7	34.2 (3.4)	29.8–39.8
Fat mass (kg) **	24.3 (4.9)	19.4–33.7	36.6 (6.2)	26.5–43.6
Lean mass (kg) **	56.2 (7.5)	45.3–68	64 (5.8)	56.4–71.2
Percent body fat	31.4 (4.3)	26.8–39	35.1 (4)	30.6–41.1
VO_2_ peak (mL/kg/min) ‡	21.8 (4.5)	17.1–27.5	23.6 (3.5)	19.6–30.2
Hand grip (kg)	35 (10.3)	23.3–51.7	38 (14.7)	19–56
1-RM leg press (lbs pressure)	442.9 (56.2)	350–500	537.5 (174.5)	350–800
Chest press (lbs pressure)	112.1 (24)	75–140	139.2 (45.1)	75–175

**Abbreviations.** BMI, body mass index; 1-RM, one-repetition maximum; VO_2_ peak, peak oxygen consumption. ‡ *n* = 6, ** *p* < 0.01. N/A—not available.

**Table 2:** Mitochondrial OCR/ECAR and protein levels.

	Mean ± SD	Range	Mean ± SD	Range
	PLWH *n* = 7		Control *n* = 7	
**Platelet OCR (pmol/min/10^7^ cells):**				
Basal	89.6 (35)	48.4–148	114.4 (36.1)	82–176.7
ATP-linked	87.7 (34.4)	46.9–144.6	98.8 (35.2)	67–155.7
Proton leak	2 (1.3)	0.2–3.8	15.6 (5.3)	9.6–21
Reserve capacity	92.4 (44.3)	34.5–150.7	76.1 (57.7)	7–173.2
Maximal	182.1 (75.6)	85.1–298.7	190.5 (92.9)	93.5–349.9
Non-mitochondrial	17 (3.9)	11–23	32.9 (9.5)	16.8–45.4
**Platelet ECAR (mpH/min/10^7^ cells):**				
Basal glycolysis	14.3 (8.4)	3.9–23.7	16.3 (3.2)	12.2–20.2
Maximal glycolysis	36.5 (16.4)	16.1–54.3	84.7 (26.7)	54.3–132.2
Glycolytic reserve	22.2 (8.4)	11.4–32.4	68.4 (25.6)	36.4–112
**Protein Levels (normalized to GAPDH):**				
SIRT3	1.41 (0.43)	0.82–2.03	___	___
COXII	0.6 (0.26)	0.31–1.04	___	___
DRP1	0.17 (0.03)	0.13–0.22	___	___
OPA1	0.19 (0.02)	0.17–0.22	___	___

**Abbreviations:** COXII, cytochrome c oxidase subunit II; DRP1, dynamin-related protein 1; GAPDH, glyceraldehyde 3-phosphate dehydrogenase; OPA1, optic atrophy protein 1; SIRT3, mitochondrial sirtuin 3.

## Data Availability

Data are available upon reasonable request.

## Abbreviations

The following abbreviations are used in this manuscript:

1-RM	1-Repetition maximum
AIDS	Acquired immunodeficiency syndrome
ATP	Adenosine triphosphate
BMI	Body mass index
cART	Combined active antiretroviral therapy
COXII	Cytochrome c oxidase subunit II
DNP	2,4 dinitrophenol
DRP1	Dynamin-related protein 1
DXA	Dual-energy X-ray absorptiometry
ECAR	Extracellular acidification rate
GAPDH	Glyceraldehyde 3-phosphate dehydrogenase
GR	Glycolytic reserve
HIV	Human immunodeficiency virus
HR	Heart rate
mtDNA	Mitochondrial DNA
NAD+	Nicotinamide adenine dinucleotide
O2	Oxygen
OCR	Oxygen consumption rate
Oligo	Oligomycin
OPA1	Optic atrophy protein 1
PBMCs	Peripheral blood mononuclear cells
PLWH	People living with HIV
PPP	Platelet-poor plasma
PRP	Platelet-rich plasma
ROS	Reactive oxygen species
SIRT3	Sirtuin 3
TCA	Tricarboxylic acid cyclecycle
VAMC	VA Medical Center
VO_2_ peak	Peak oxygen consumption
